# PSCA-directed nanosized bio-immune conjugates (NANO:BICs) enable selective uptake of TLR9 agonists in bladder cancer cells

**DOI:** 10.3389/fonc.2026.1739618

**Published:** 2026-04-28

**Authors:** Max Iltzsche, Nancy Wetterling, Lissy Jilek, Daniel Nahhas, Marlena Hesse, Stefanie Tietze, Achim Temme, Christian Thomas, Susanne Fuessel, Barbara Kind

**Affiliations:** 1 Department of Urology, Medical Faculty Carl Gustav Carus, Technische Universität Dresden, Dresden, Germany; 2 Department of Neurosurgery, Section Experimental Neurosurgery and Tumor Immunology, Medical Faculty Carl Gustav Carus, Technische Universität Dresden, Dresden, Germany; 3 German Cancer Consortium (DKTK), Partner Site Dresden, Dresden, Germany; 4 National Center for Tumor Diseases (NCT), German Cancer Research Center (DKFZ), Faculty of Medicine and University Hospital Carl Gustav Carus, Technische Universität Dresden, Helmholtz-Zentrum Dresden-Rossendorf (HZDR), Heidelberg, Germany

**Keywords:** bladder cancer, fluorescence intensity analysis, immunotherapy, NANO:BICs, NMIBC, PSCA, single-chain antibodies, targeted therapy

## Abstract

**Introduction:**

Bladder cancer (BCa), particularly non-muscle invasive bladder cancer (NMIBC), remains a significant healthcare challenge due to high recurrence rates and limited non-surgical treatment options.

**Methods:**

Prostate stem cell antigen (PSCA)-transduced HEK-BlueTMhTLR9 and PSCA-positive SW780 bladder cancer cells were stimulated with PSCA-targeting NANO:BICs, which were assembled from a scFv(AM1)-KiBAP, NeutrAvidin, and the Toll-like Receptor 9 (TLR9) agonist ODN2006. Functional analyses included a secreted embryonic alkaline phosphatase (SEAP) reporter assay to measure TLR9 activation, a Cytometric Bead Array for cytokine quantification, and confocal microscopy to assess cellular uptake.

**Results:**

PSCA-targeting NANO:BICs demonstrated significantly enhanced uptake into PSCA-positive cancer cells compared to non-targeting controls, with the PSCA receptor increasing uptake by a factor of 3.63. This targeted delivery led to potent activation of the TLR9 signaling pathway, evidenced by a robust reporter gene response and secretion of key antiviral cytokines, including type I and III interferons and the chemokine IP-10.

**Discussion:**

These findings highlight the potential of this approach not only for reinvigorating anti-tumor immune responses in BCa but also for broader applications in other PSCA-expressing malignancies.

## Introduction

**Figure 1:**
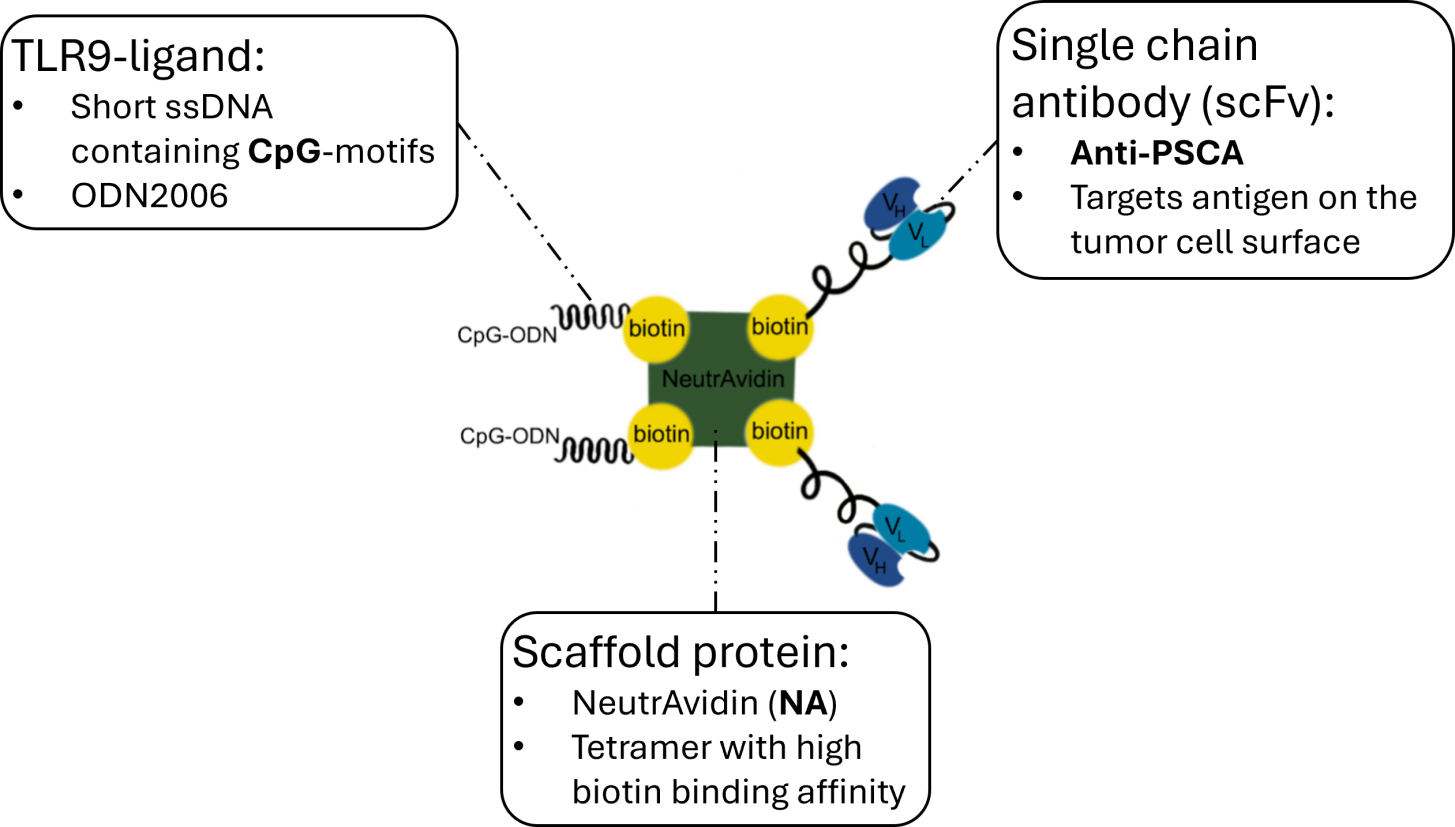
Schematic composition of NANO:BICs. The figure illustrates the assumed molecular construct of NANO:BICs, combining a scaffold protein, a TLR9 ligand, and a single-chain antibody. The scaffold protein, NeutrAvidin (NA), served as the central component with high biotin-binding affinity, allowing it to anchor both biotinylated TLR9 ligands and single-chain antibodies. The TLR9 ligand was a single-stranded DNA (ssDNA) containing CpG motifs, such as ODN2006, which were recognized by TLR9 receptors of cells, triggering an immune response. The single-chain antibody, anti-PSCA, specifically targeted the PSCA antigen on tumor cells, ensuring that the construct can bind to the tumor site. This design is expected to enable localized immune activation, where the CpG-motifs can activate immune cells near the tumor, promoting an anti-tumor response. Instead of the PSCA-specific scFv(AM1)-KiBAP, control NANO:BICs were assembled using an anti-EGFRvIII (scFv(MR1.1)-KiBAP), which, due to EGFRvIII deficiency of the cells, did not target antigens on the examined cells. This approach aimed to improve the specificity and efficacy of cancer immunotherapy by combining precise tumor targeting with potent immune activation.

The current standard of care for high-risk non-muscle invasive bladder cancer (NMIBC) is intravesical immunotherapy with live Bacillus Calmette-Guérin (BCG), which has remained the most effective agent for reducing tumor recurrence and progression for decades (1–3). Its therapeutic effect is driven by a complex, non-specific inflammatory response initiated through the activation of pattern recognition receptors like TLR2 and TLR4 on both urothelial and immune cells (4). However, despite its proven efficacy, BCG therapy is beset by significant limitations that create a pressing need for novel therapeutic strategies. These challenges include a high failure rate, with 30-50% of patients being or becoming unresponsive, and significant toxicity, where the potent immune activation frequently causes debilitating side effects that can require cessation of treatment (1, 3–5). Compounding these clinical issues, global manufacturing problems have led to worldwide shortages of BCG, further limiting patient access (3). These limitations highlight the need for new immunotherapies that can match or exceed BCG’s efficacy while offering a superior safety profile. The mechanism of BCG itself suggests that targeted activation of specific immune pathways, such as with TLR agonists, could be a promising approach (4, 6). Our NANO: BIC platform (**Figure 1**) is designed to address the shortcomings of BCG directly by using a nanoparticle to deliver a specific TLR9 agonist to PSCA-expressing cancer cells. This strategy aims to replicate the beneficial immunostimulatory effects of BCG but with the key advantages of enhanced specificity, by activating a single TLR pathway, and targeted delivery, which concentrates the therapeutic agent at the tumor site to maximize efficacy and minimize systemic side effects. In doing so, NANO:BICs could represent a next-generation, precision immunotherapy with the potential to improve upon the current standard of care for NMIBC. In this study, we demonstrate the successful application of nanosized bio-immune conjugates (NANO:BICs) for the selective delivery of CpG-oligodeoxynucleotides (ODN), potent synthetic agonists for endosomal TLR9, to prostate stem cell antigen (PSCA)-positive bladder cancer (BCa) cells. PSCA, frequently expressed in urological cancers, represents a promising therapeutic target (7–9). The NANO:BICs, equipped with a single-chain antibody (scFv) against PSCA, facilitated receptor-mediated endocytosis and enabled the activation of endosomal TLR9, which is known to trigger anti-tumor immune responses (10). Previous studies have shown that similar bio-immune conjugates, loaded with TLR3 agonists, induced type-I interferon responses and apoptosis in target cells, earning the synonym Rapid Inducer of Cellular Inflammation and Apoptosis (RICIA) (11).

Fluorescent nanoparticles are essential tools for studying intracellular uptake and trafficking, offering precise insights into the movement and localization of molecules, vesicles, and organelles, which are critical for understanding processes like endocytosis, exocytosis, and autophagy (12). Disruptions in these processes are linked to diseases such as neurodegeneration, cancer, and infections (13). Additionally, fluorescent tracking has proven invaluable for optimizing drug delivery systems by confirming the targeted delivery of therapeutic agents (14). Using automated fluorescence microscopy analysis, we confirmed the selective uptake of NANO:BICs via the PSCA receptor in both reporter and BCa cell lines. These findings validate the targeting function of the anti-PSCA scFv and highlight PSCA as a viable target for NANO:BIC-mediated delivery in BCa models. Moreover, this study provides a valuable tool for evaluating targeted delivery systems through automated fluorescence microscopy analysis.

## Materials and methods

### Cell culture

Human HEK-Blue™hTLR9 (Invivogen) and HEK-Blue™hTLR9-PSCA cell lines were cultured in Dulbecco’s Modified Eagle Medium (DMEM) complemented with 4.5 g/l glucose, 10% v/v heat-inactivated fetal bovine serum (FBS), 0,1% v/v blasticidin and 0,1% v/v zeocin (all from Thermo Fisher Scientific). For better reading, the cells are denoted as “HekBlue” in the figures. The BCa cell line SW780 (ATCC), derived from a grade 1 BCa, with endogenous expression of PSCA was cultured in DMEM complemented with 4.5 g/l glucose supplemented with 10% v/v heat-inactivated FBS (15). All cell lines were incubated at 37 °C and 5% CO_2_ in a humidified incubator.

### Lentiviral transduction

PSCA was introduced into HEK-Blue™hTLR9 cells to generate HEK-Blue™hTLR9-PSCA cells as a suitable model for NANO:BICs-mediated targeting. Transduction of the PSCA coding sequence was accomplished as described previously (16). Briefly, cells were transiently transfected with the packaging vectors pCD/NL-BH (17, 18) as well as the lentiviral transfer vector p6NST53-PSCA using polyethyleneimine (PEI) (Polysciences). Production of viral particles was enhanced with 10 µM sodium butyrate (Sigma-Aldrich) 12 h after transfection. Medium was changed after 8 h and lentiviral supernatant was harvested 12 h later, filtered with a 0.45 µm pore size filter and supplemented with 8 µg/ml polybrene (Sigma-Aldrich). Transduction of 1.5 × 10^5^ HEK-Blue™hTLR9 cells was performed in a 6-well with 2 ml of lentiviral supernatant and repeated after 24 h. The resulting transduced HEK-Blue™hTLR9-PSCA cells were selected with 0.5 mg/ml geneticin (Invitrogen) for 24 h.

### NANO:BICs-assembly

Prior to the assembly of NANO:BICs, a pre-complex of the biotinylated scFv and NeutrAvidin (Thermo Fisher Scientific; 60 kDa) was conjugated for 30 min in phosphate buffered saline (PBS) at room temperature (RT). For NANO:BICs targeting PSCA the scFv(AM1)-KiBAP (40.4 kDa) was used (11). For control NANO:BICs the biotinylated scFv(MR1.1)-KiBAP targeting epidermal growth factor receptor variant III (EGFRvIII; 36.6 kDa), a mutation rarely found in BCa (19), was used (11). Our in-house “KiBAP” sequence corresponded to the AviTag. The scFv modulated with the AviTag are referred to with the suffix “KiBAP” in the figures. The AviTag is a 15-amino acid Biotin Acceptor Peptide (BAP) with the sequence GLNDIFEAQKIEWHE (20). Its site-specific biotinylation allowed for the highly efficient and oriented conjugation of our targeting moiety to the avidin-core of our nanoparticles. Recombinant scFv-KiBAPs were produced as described recently (11). The fluorescently labelled and mono-biotinylated TLR9 agonist ODN2006 (21) with a full phosphorothioate (PTO) backbone (TIB Molbiol) was then added to the pre-complex and incubated at room temperature for 30 min in a final molar ratio of 1:1:2 (scFv 0.25 µM: NeutrAvidin 0.25 µM: ODN2006 0.5 µM). The medium used for the cultivation of the respective cell line to be incubated with the NANO:BICs was added to the assembled complexes. The medium was supplemented with 2.5% v/v heat-inactivated FBS. Cells grown to 70-80% confluence in Falcon 8 chamber slides (Corning) were stimulated with 100 µl of this NANO:BICs-solution or only ODN in the same concentration used for NANO:BICs for 24 h at 37 °C and 5% CO_2_ before immunofluorescence staining. Controls were treated with 100 µl of the respective medium (2.5% heat-inactivated FBS).

### Cell stimulation

For stimulation with NANO:BICs cells were grown to 40 – 50% density after seeding on 96-well microplates (Corning) and stimulated with the NANO:BICs dissolved in DMEM thereafter. Cells treated with their corresponding medium served as a negative control. After 72 h incubation at 37 °C, the cells were stimulated. 4 h and 24 h after stimulation, 100 µl of the cell culture supernatants were removed and the cytokine concentration was determined using a cytometric bead assay.

### Cytometric bead array

Cells were seeded in 96-well microplates and incubated for 72 h. The cells were stimulated and 4 h and 24 h after stimulation, 100 µl of the cell culture supernatants were removed and the cytokine concentration was determined. For quantitative analysis of cytokines in the cell culture supernatants a LEGENDplex™-Assay human Anti-Virus Panel (BioLegend) was performed according to the manufacturer’s manual. This panel allows simultaneous quantification of 13 human proteins, with interferons, IP10 and pro-inflammatory interleukins among them. The raw data of the flow cytometric measurements acquired by MACSQuant Analyzer 10 (Miltenyi Biotec) was analyzed with the web-based application Qognit (BioLegend).

### Quanti-Blue Assay

The QBA (Invivoqen) represents a reporter assay for the colorimetric detection of secreted embryonic alkaline phosphatase (SEAP activity) in stimulated HEK-Blue*™*hTLR9-PSCA cells. The cells were seeded and stimulated in conventional cell culture medium. The reporter assay was then performed using 20 µl of cell culture supernatant, which was added to 180 µl of Quanti Blue ™ solution in a 96-well microtiter plate (Corning). This was followed by incubation at 37 °C for 1 h. Activation of the reporter system resulted in a color change from magenta to blue, which was quantitatively determined by measuring the absorption at 620 nm using the microtiter plate reader Mithras LB 940 (Berthold Technologies). As a blank for the measurement, 20 µl of pure cell culture medium without cells, which were incubated in the same plate as the stimulated cells, were used together with 180 µl of sonst Quanti (klein geschrieben) and also incubated for 1 hour at 37 °C.

### Immunofluorescence staining

For immunofluorescence experiments, 8,750 cells per well were seeded on Falcon 8 chamber slides (Corning). After growth to 70-80% confluence the NANO:BICs and controls were added to the cells and incubated at 37 °C and 5% CO_2_ for 24 h. The medium of the respective cell lines was then removed, and the cells were washed twice with 600 µl PBS. Cells were then fixed with 4% paraformaldehyde for 20 min and washed three times with PBS for 5 min each. All incubation steps were performed at room temperature in the dark. For staining with Wheat Germ Agglutinin (WGA) membrane dye conjugated to Alexa Fluor^®^ 647 fluorophore (Thermo Fisher Scientific), cells were incubated for 20 min in 200 µl dye in 1:200 Hank’s Balanced Salt Solution (HBSS; Thermo Fisher). Cells were then washed twice with HBSS. For the staining of intracellular proteins, cells were previously permeabilized with 0.2% Triton X-100 (Sigma-Aldrich) for 10 min and washed with 1% bovine serum albumin (BSA; Serva Electrophoresis) in HBSS. The mounting medium Vectashield (BIOZOL) containing the cell nuclear stain 4′,6-diamidino-2-phenylindole (DAPI) was added to the wells in a volume of 50 µl each and the slide was then covered with a coverslip. The next day, the edges were sealed with nail polish and the slide was stored at 4 °C for further use.

### Confocal laser scanning microscopy

Imaging experiments were performed using a Zeiss LSM 880 confocal laser scanning microscope with Airyscan detector at the Core Facility Cellular Imaging at the Medical Faculty Carl Gustav Carus Dresden. Image J software was used to analyze the microscopy images. For this purpose, the original files were opened in the Image J software and separated into its channels to distinguish the different components within the samples. The channels corresponded to different stains, each highlighting specific structures, either of the cells themselves or of NANO:BICs. Afterwards these separate channels of the original file were split into individual images creating three distinct images. To visualize cell nuclei DAPI-staining was used. The DAPI fluorescent stain was detected in the blue channel at 457 nm. WGA Alexa Fluor^®^ 647 Conjugate was used to label cell membranes and detected in the red channel at 647 nm. The commonly used fluorescent label FITC was used in conjugation with the ODN as a labeled NANO:BICs component and detected in the green channel at 488 nm. A Gaussian filter was applied to the DAPI channel to counteract and reduce background noise. Using selected automatic thresholds, the channel was binarized. This automatic thresholding technique was applied to each image to segment the stained regions from the background. Applied specifically to the DAPI-staining image, the watershed function was used to separate or delineate touching or overlapping objects (in this case cell nuclei) within the image. Using the Analyze Particles command (Size 50-Infinity; Exclude on edges; Summarize) the nuclei were quantified to determine the number of cells in the image. A Gaussian filter was applied to the WGA channel and the channel was binarized thereafter. This was done using an auto-threshold algorithm previously reviewed and determined on exemplary images. The thresholds used were automatic algorithms integrated into the Image J software (22). The binarized channel was used to create selection. Since WGA stains the cell surface, this selection was indicative of the cell surface area, which was stored as a region of interest (ROI). The ROI created was now applied to the FITC channel, allowing the following measure to be limited to the cells surface area. The selected region of the FITC channel was then analyzed (Measure). In addition to the analyzed area, the mean gray value and its product with the area (IntegratedDensity (IntDen)) were determined to enable the measurement of fluorescence intensity or other relevant metrics within the selected regions (e.g., intensity of the green fluorescence within the areas defined by the cell membrane). Thus, it was possible to determine values for the intensity of the FITC channel only in the areas of cells (excluding the background) by utilizing the ROI derived from the WGA channel. The integrated density was then divided by the number of cells in the DAPI channel of the same image to obtain a standardization by cell size (IntDen/Cell). A self-written script was used to automate the analysis process (see **Supplementary Methods**).

### Statistical analysis

Statistical analysis was performed using Prism software (Version 10, GraphPad Software Inc). To assess the statistical significance of differences between two independent experimental groups, the non-parametric Mann-Whitney U test was employed. This test was chosen because, as a rank-based method, it does not assume a normal distribution of the data, making it appropriate for the analysis of our experimental results. For all analyses, a two-tailed test was performed, and a p-value of less than 0.05 was considered statistically significant. In the figures, brackets are used to denote the specific groups being compared. The corresponding p-value thresholds are indicated above the brackets using the following convention: p > 0.05 (ns, not significant); p ≤ 0.05 (*); p ≤ 0.01 (**); p ≤ 0.001 (***); and p ≤ 0.0001 (****).

## Results

**Figure 2:**
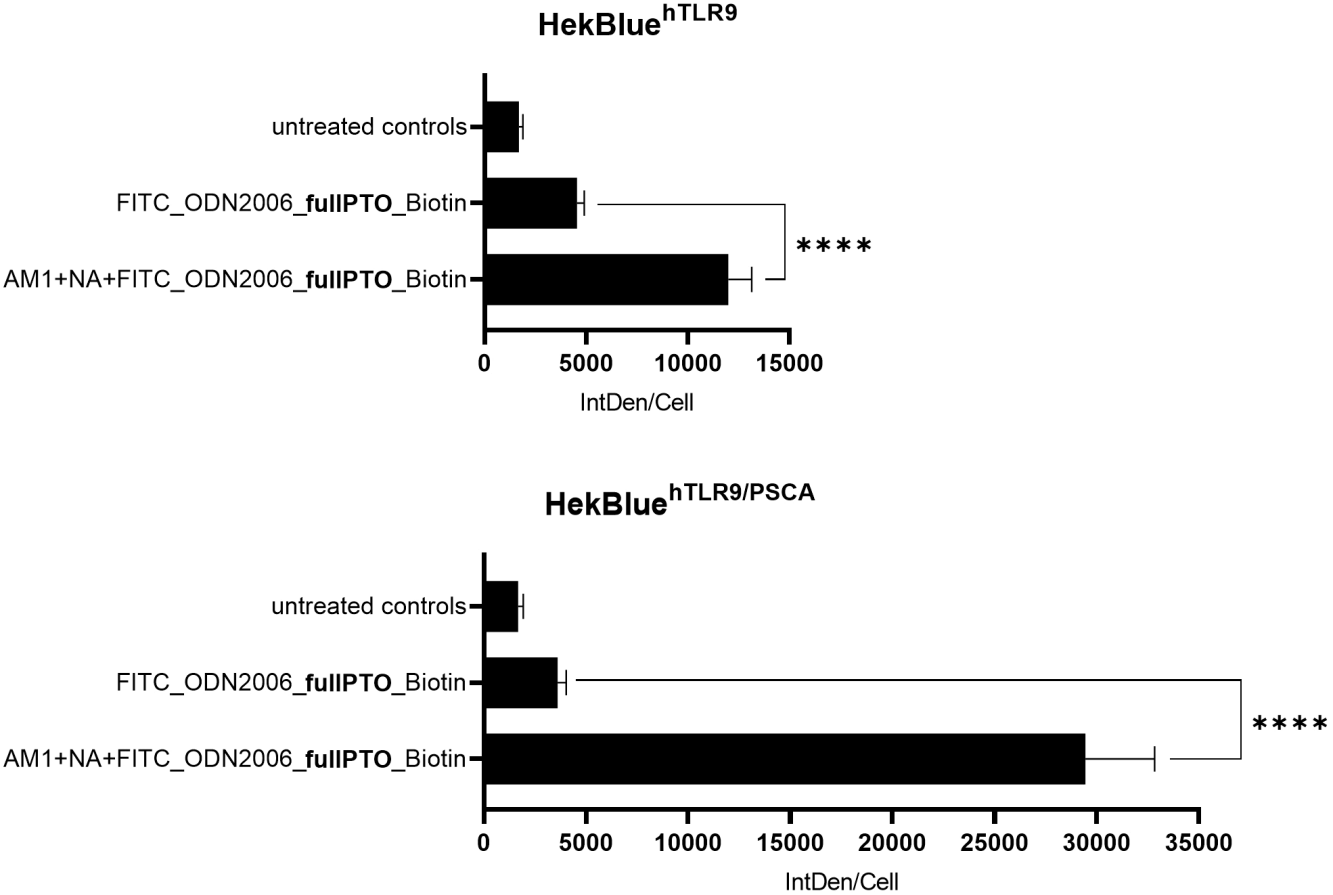
Uptake of different NANO:BICs treatments by the cell lines HEK-Blue™hTLR9 and HEK-Blue™hTLR9-PSCA. Cells were seeded in an 8-well culture slide. After 72 h incubation in an incubator at 37 °C, the cells were incubated with fluorescently labeled NANO:BICs or ODN. 24 h after the start of stimulation, the cells were fixed. Cell components were stained after fixation and microscoped together with the stained NANO:BICs. The intensity measured over the area of the cells in an image section was divided by the number of cells present in this image section for standardization. For better comparison, the mean of the untreated controls in one series of experiments were calculated and used for normalization. N = 20; MW ± SD; For all analyses, a two-tailed test was performed, and a p-value of less than 0.05 was considered statistically significant. In the figures, brackets are used to denote the specific groups being compared. The corresponding p-value thresholds are indicated above the brackets using the following convention: p ≤ 0.0001 (****).

The NANO:BICs target delivery system used in the project had a modular composition (**Figure 2**). A central NeutrAvidin (NA) served as a scaffold structure. Biotinylated TLR agonistic ODN and biotinylated scFv directed against a surface receptor of BCa cells - PSCA were bound to it via non-covalent bonds. The bond was formed between the NA, which consisted of four identical subunits, each with a binding site, and a biotin molecule conjugated to the individual components. Synthetically produced nucleic acids - ODN - with CpG dinucleotides were used as specific TLR agonists. A comprehensive characterization of the NANO:BICs was conducted prior to performing the subsequent experiments (**Supplementary Methods** and **Supplementary Figure 1**, **Supplementary Figure 2**, **Supplementary Figure 3**).

Flow cytometry was used (as explained in the **Supplementary Methods** section) to assess PSCA and EGFRvIII expression in BCa and reporter cell lines (**Supplementary Figure 4**, **Supplementary Figure 5**). Among the analyzed cell lines, HEK-Blue™hTLR9 and HEK-Blue™hTLR9-PSCA were used as reporter cell lines, while the SW780 cell line served as a cancer model cell line. In the histograms, green curves represent cells with the receptor fluorescently stained, with greater curve shifts indicating higher receptor expression. For the SW780 cell line, PSCA was detected on 91.3% of cells, whereas EGFRvIII expression was minimal (0.78%) (**Supplementary Figure 4**). These results confirmed PSCA as a surface target for NANO:BICs in SW780 cells. On the other hand, the scFv(MR1.1)-KiBAP failed to bind to SW780 cells and, therefore, represented an appropriate control antibody to investigate unspecific uptake of NANO:BICs mediated by NA and CpG-ODN components. PSCA was detected in 99.9% of HEK-Blue™hTLR9-PSCA cells and was not found in HEK-Blue™hTLR9 cells (**Supplementary Figure 5**). Flow cytometry showed no convincing increase in fluorescence over the control. 4.1% of cells were scored as positive by gating but without a discernible histogram shift. The findings above confirmed PSCA as a target for NANO:BICs in HEK-Blue™hTLR9-PSCA and SW780 cells and established a model for comparing NANO:BIC uptake based on PSCA expression.

**Figure 3:**
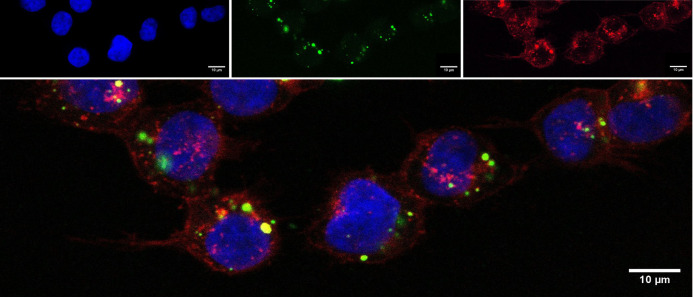
Fluorescence labeled cells of the cell line SW780 and channels of separately stained components (top) and merged image (bottom). For visualization of cell nuclei DAPI-staining was used. The DAPI fluorescent stain was detected in the blue channel at 457 nm. The fluorescent label FITC was used in conjugation with the ODN as a labeled NANO:BICs component and detected in the green channel at 488 nm. WGA was used to label cell membranes and detected in the red channel at 647 nm.

The cells fluorescently labeled with NANO:BICs were analyzed using fluorescence microscopy. The localization of the analyzed particle or cell component was assessed by staining the cell membranes and the DNA in the nucleus and superimposing the individual images (**Figure 3**).

**Figure 4:**
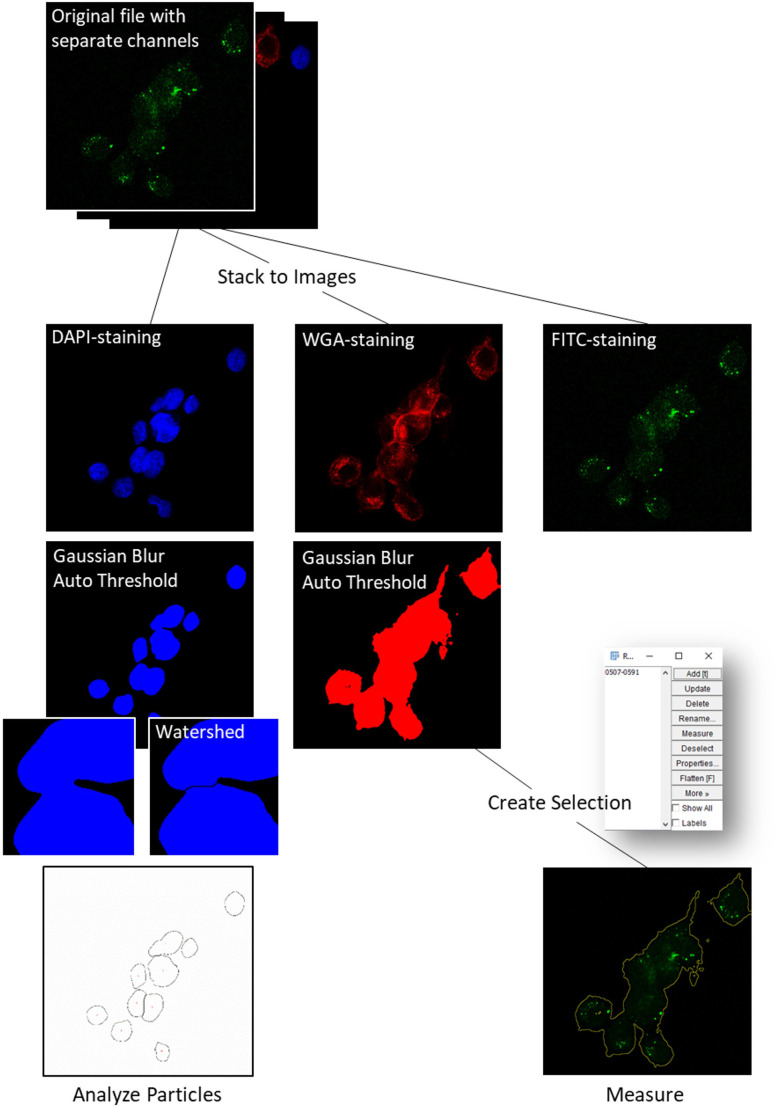
Flow chart of the method of analysis employed for fluorescence microscopy images. A description of the steps taken to process the images can be found in the methods section.

For the investigation of uptake of NANO:BICs into PSCA-positive cells of the respective cell lines, cells were treated with fluorescently marked NANO:BICs and examined under a microscope. The images acquired using fluorescence microscopy were analyzed using a dedicated script for the Image J software (**Figure 4**).

**Figure 5:**
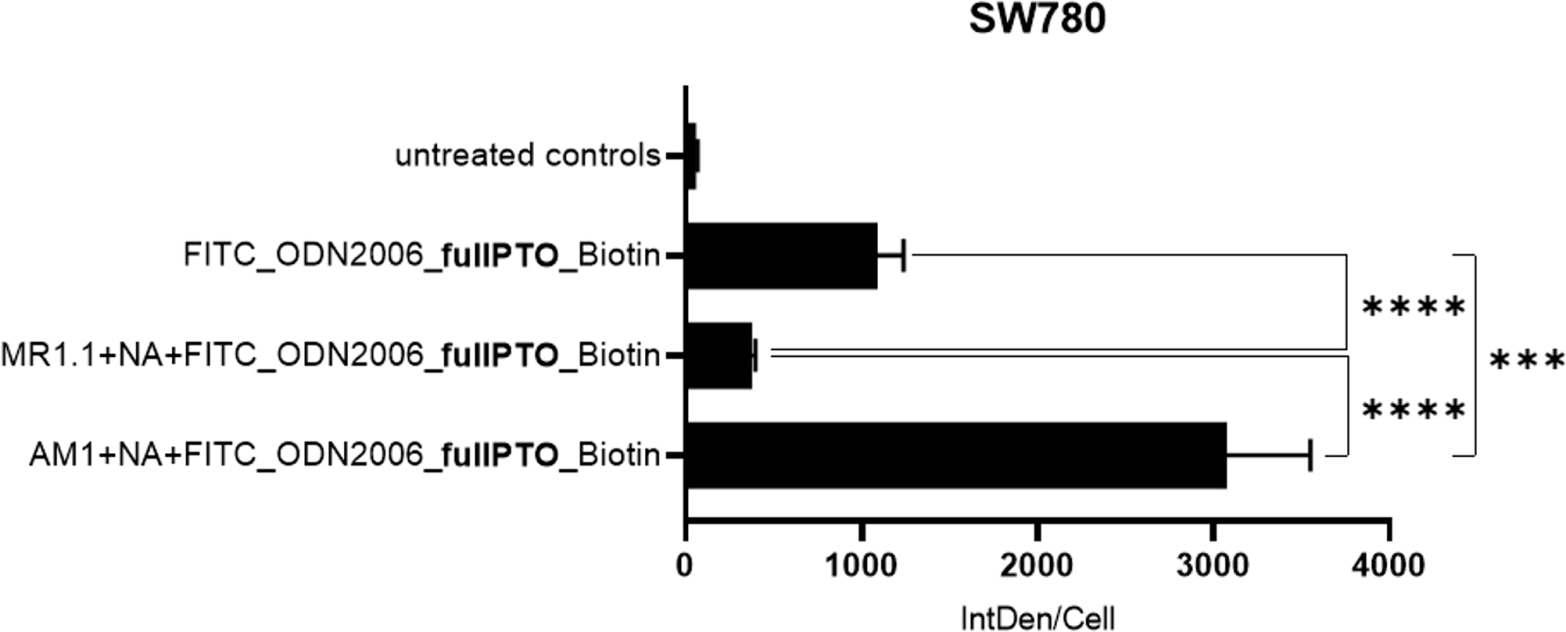
Uptake of the NANO:BICs and ODN in different treatments of the SW780 cell line. The cells were seeded in an 8-well culture slide at a cell number of 8750 cells per well. After 72 h incubation in an incubator at 37 °C, the cells were incubated with fluorescently labeled NANO:BICs or ODN. 24 h after the start of stimulation, the cells were fixed. Cell components were stained after fixation and microscoped together with the stained NANO:BICs. The graph measures the integrated density (IntDen) per cell as a quantitative metric used to assess fluorescence intensity. This indicated the level of the fluorescent marker FITC in each cell as the intensity measured over the area of the cells in an image was divided by the number of cells present in this image for standardization. N = 20; MW ± SD; For all analyses, a two-tailed test was performed, and a p-value of less than 0.05 was considered statistically significant. In the figure, brackets are used to denote the specific groups being compared. The corresponding p-value thresholds are indicated above the brackets using the following convention: p ≤ 0.001 (***); and p ≤ 0.0001 (****).

The untreated control group showed minimal IntDen/Cell as a quantitative metric used to assess fluorescence intensity, suggesting a low baseline fluorescence, as expected (**Figure 5**). This baseline fluorescence was most likely caused by the autofluorescence of the cells as the intensity was only measured over the cells surface areas as explained in the methods section. Cells treated only with the ODN (FITC_ODN2006_fullPTO_Biotin) showed an increase in intensity signal compared to the untreated controls indicating an uptake into the cells. The cells treated with NANO:BICs with the PSCA-specific antibody (AM1+NA+FITC_ODN2006_fullPTO_Biotin) showed the highest fluorescence signal and thus the best uptake. NANO:BICs with the PSCA-specific scFv and ODN (AM1+NA+FITC_ODN2006_fullPTO_Biotin) were taken up significantly better than NANO:BICs with the non-specific control scFv and ODN (MR1.1+NA+FITC_ODN2006_fullPTO_Biotin) and the ODN itself.

In an experiment with the reporter cell lines HEK-Blue™hTLR9 and HEK-Blue™hTLR9-PSCA, the uptake of NANO:BICs and ODN was compared (**Figure 1**). The HEK-Blue™hTLR9 cells did not express PSCA (as shown in **Supplementary Figure 5**), meaning they had no target for the PSCA-specific scFv(AM1)-KiBAP of the NANO:BICs. Thus, the effect of the scFv on the uptake of the NANO:BICs into the cells could be investigated. Some non-specific uptake of PSCA-targeting NANO:BICs in PSCA-negative HEK-Blue™hTLR9 cells was observed.

Both cell lines HEK-Blue™hTLR9 and HEK-Blue™hTLR9-PSCA showed a significant increase in fluorescence intensity when treated with ODN alone (FITC_ODN2006_fullPTO_Biotin) compared to the untreated controls. The ratio of the intensity per cell of NANO:BICs (AM1+NA+FITC_ODN2006_fullPTO_Biotin) to ODN (FITC_ODN2006_fullPTO_Biotin) was 2.24 for the HEK-Blue™hTLR9 cell line. Thus, NANO:BICs were taken up approximately twice as well as the single ODN. Treatment of HEK-Blue™hTLR9-PSCA cells yielded similar pattern and resulted in similar conclusions (**Figure 1**). The cells took up NANO:BICs with a specific antibody targeting the cells PSCA (AM1+NA+FITC_ODN2006_fullPTO_Biotin) best. For the HEK-Blue™hTLR9-PSCA cell line, the ratio was 8.15. Thus, uptake was increased by 3.63 times by the presence of the surface receptor to which the scFv of the NANO:BICs could bind.

**Figure 6:**
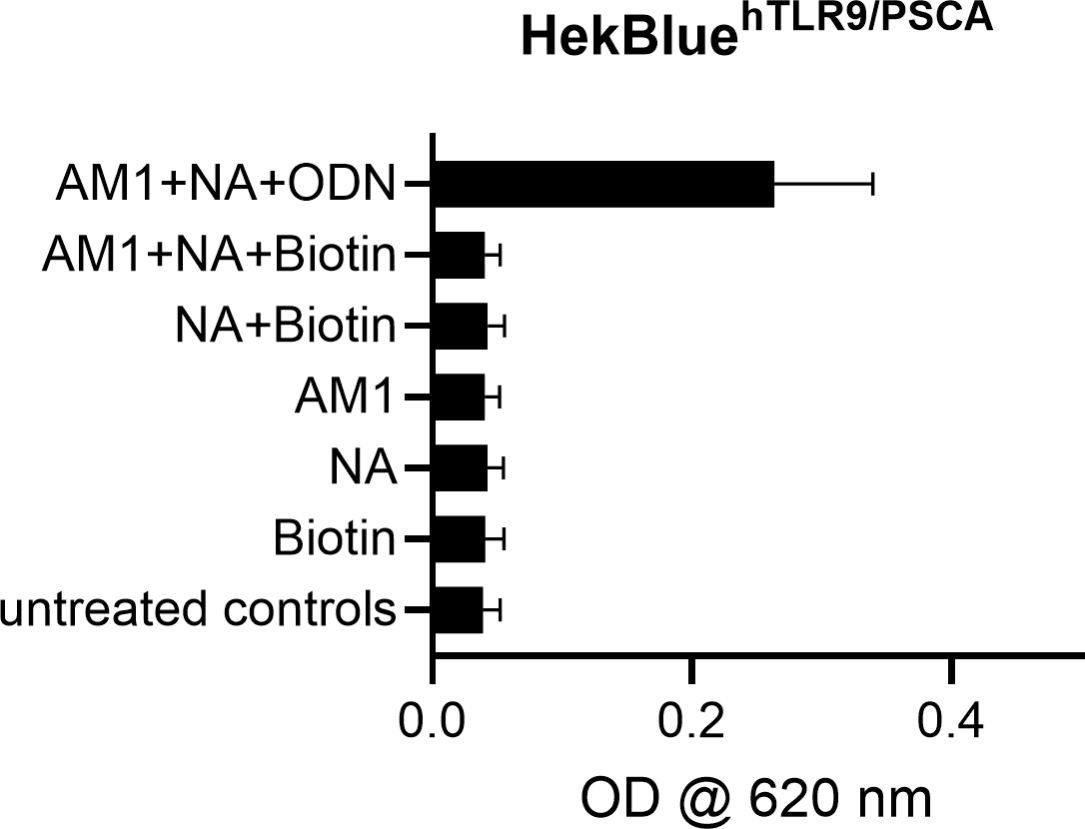
Confirmation of hTLR9 activation using QBA 24 h after Nano: BICs stimulation. HEK-Blue™hTLR9-PSCA cells were stimulated for 24 h with different Nano:BIC variants and individual components. hTLR9 activation was monitored using QBA. The figures show the mean values (bars) ± SEM from three independent experiments with double determination for each stimulation.

TLR9 activation by NANO:BIC was first assessed using a Quanti-Blue™ reporter assay in HEK-Blue™ hTLR9-PSCA cells 24 h after stimulation (**Figure 6**). A robust increase in SEAP activity was observed upon stimulation with the PSCA-targeting TLR9 agonist containing formulation, indicating strong activation of TLR9-dependent NF-κB/AP-1 signaling. In contrast, control formulations lacking the CpG-ODN component (AM1 + NA + Biotin, NA + Biotin, AM1 alone, NA alone, Biotin alone) induced only background-level responses comparable to untreated controls.

**Figure 7:**
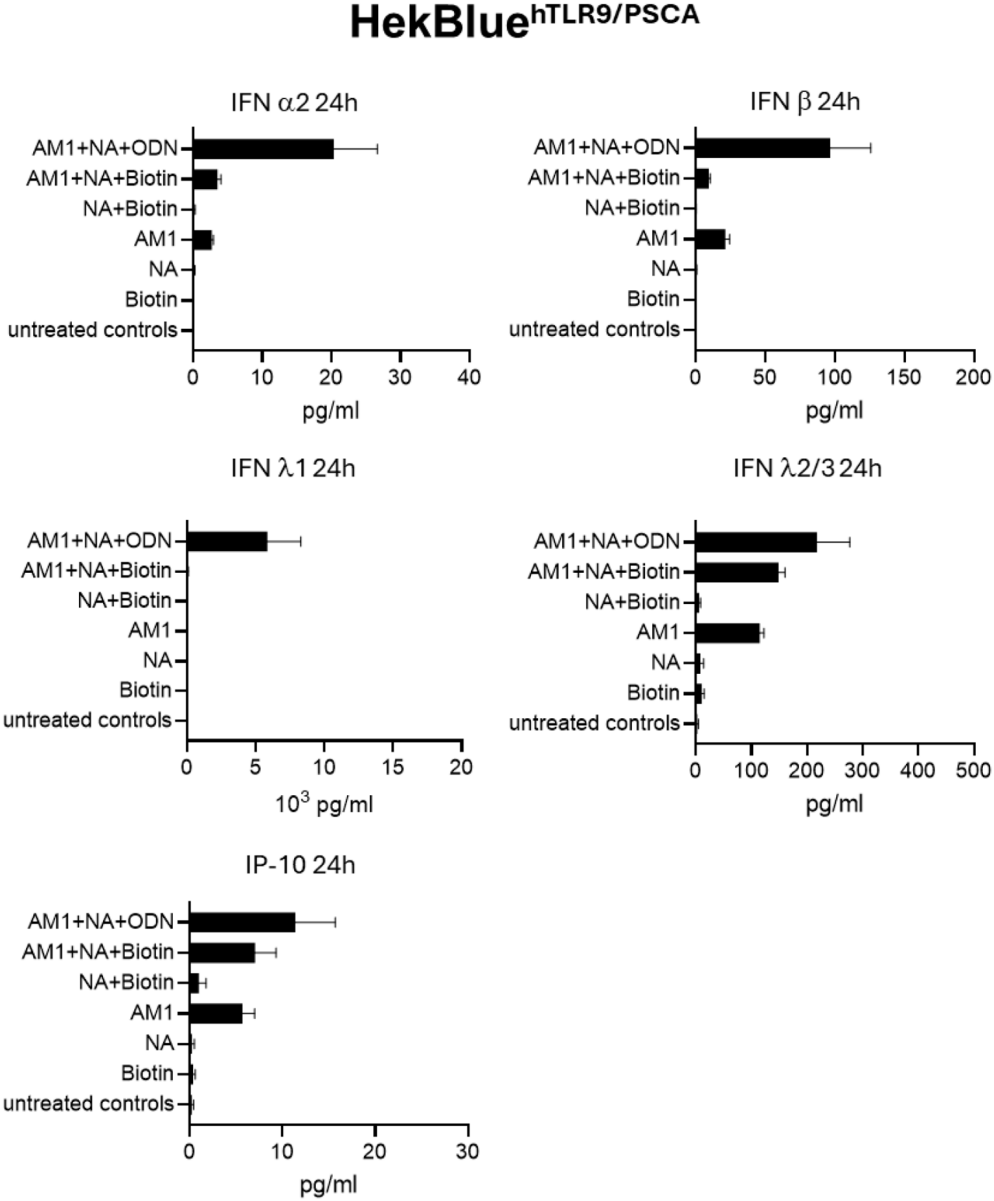
Confirmation of hTLR9 activation observing the secretion of cytokines 24 h after stimulation of HEK-Blue™hTLR9-PSCA cells with NANO:BICs. HEK-Blue™hTLR9-PSCA cells were stimulated for 24 h with different NANO: BIC variants and individual components. The secretion of cytokines was monitored using LEGENDplex™ multiplex immunoassay. The figures show the mean values (bars) ± SEM from three independent experiments with double determination for each stimulation.

To confirm functional downstream signaling of TLR9 activation after NANO:BIC stimulation, cytokine secretion profiles were analyzed by LEGENDplex™ multiplex immunoassay 24 h after stimulation (**Figure 7**). Consistent with the Quanti-Blue™ data, AM1 + NA + ODN induced pronounced secretion of type I and type III interferons, including IFN-α2, IFN-β, IFN-λ1, and IFN-λ2/3, as well as the TLR9-associated chemokine IP-10.

## Discussion

This study demonstrated the successful intracellular uptake of NANO:BICs via receptor-mediated internalization in PSCA-positive BCa cells. Using fluorescence microscopy, we confirmed that NANO:BICs equipped with the PSCA-specific scFv(AM1)-KiBAP achieved significantly higher uptake in PSCA-positive reporter cells and the human BCa cell line SW780 compared to control NANO:BICs containing the non-specific anti-EGFRIII antibody. These findings confirmed the specificity of the scFv(AM1)-KiBAP for PSCA-positive cells, as previously reported (23), and highlight its potential for targeted therapeutic delivery.

Quantitative analysis revealed that the presence of the PSCA-specific scFv significantly enhanced nanoparticle uptake, with a nearly fourfold improvement compared to non-specific controls. This superior uptake was observed across all PSCA-positive cell lines tested and was consistent with the hypothesized mechanism of receptor-mediated endocytosis. Importantly, control experiments with the scFv(MR1.1)-KiBAP, which targets the mutated EGFRvIII (a mutation rarely found in BCa; 19), confirmed the specificity of the PSCA-targeting approach. While some non-specific uptake of NANO:BICs was observed, it was significantly lower than the uptake mediated by the PSCA-specific scFv, further supporting the role of PSCA as the primary target. The underlying mechanism causing some non-specific uptake of PSCA-specific NANO:BICs in PSCA-negative cells, could be related to the PTO backbone of the ODNs, yet should bear no risks of adverse effects when given in orthotopic applications such as intravesical instillation for treatment of BCa. On the other hand, systemic treatment with PTO ODN-loaded NANO:BICs to treat other types of cancer might induce platelets activation via interacting with the collagen receptor GPVI and, therefore, might require non-modified ODNs (24).

The automated fluorescence microscopy analysis developed in this study proved to be a robust and reproducible method for quantifying nanoparticle uptake. By employing automated thresholding algorithms in Image J (22), we minimized observer variability and ensured consistent measurements across experiments. These were selected based on various sample images to optimize the selection of the cell membrane allowing for a sample-tailored approach. However, the use of an algorithm does not preclude errors in the labeling of the cell membrane – the algorithm merely performs operations on image data predefined within it. Therefore, images with inaccurately labeled cell membranes had to be sorted out during inspection (of images of the threshold separately saved by the script). This method also allowed for the exclusion of background fluorescence and inaccurate cell membrane labeling, enhancing the reliability of the results. Nevertheless, the use of an algorithm enabled the reproducibility of measurements while minimizing intra- and inter-observer variability. However, adjustment of the automatic threshold to different conditions or its replacement may still be feasible when applying the method to different cell lines.

The reporter gene activation and cytokine profiling provide convergent and direct evidence for specific activation of the TLR9 pathway by CpG-ODN–containing constructs. First, the transcriptional activity (SEAP) confirmed that the TLR9-induced NF-κB signaling cascade was successfully engaged. Second, the translational output (cytokine secretion) demonstrated that this activation led to an inflammatory response characterized by a potent Type I/III interferon signature. Control NANO:BICs without ODN showed minimal cytokine induction, confirming that nanoparticle components alone are insufficient to trigger the observed immune activation. The results align with previous findings by Schau et al. (11), who demonstrated the potential of NANO:BICs as a modular system (RICIA) for delivering TLR3 agonists to PSCA-positive tumor cells, inducing type I interferon responses and apoptosis. Our study expands on these findings by confirming the specificity of NANO:BICs for PSCA-positive BCa cells and providing a quantitative framework for analyzing their uptake. Additionally, the observed uptake ratios underscore the critical role of the scFv component in facilitating targeted internalization.

Receptor-mediated uptake mechanisms, such as those demonstrated here, have broad implications for drug delivery and therapeutic applications. Similar strategies have been employed using scavenger receptors for oligonucleotide-functionalized nanoparticles (25) and transferrin receptors for site-specific delivery of anticancer agents (26). Functionalized gold nanoparticles have also been shown to exploit receptor-mediated endocytosis for targeted delivery in breast cancer cells (27). These studies, together with our findings, emphasize the importance of receptor-specific targeting in advancing nanoparticle-based therapies.

In conclusion, this study highlights the specificity and efficiency of PSCA-targeted NANO:BICs for delivering phosphothioate-modified CpG-ODNs to PSCA-positive BCa cells. The developed fluorescence-based analysis method provides a valuable tool for evaluating receptor-mediated nanoparticle uptake and supports the broader application of NANO:BICs in therapeutic strategies targeting PSCA and other tumor-specific antigens.

Building on this proof-of-concept, future work will focus on translating NANO:BICs into a therapeutic strategy through comprehensive *in vivo* studies. Orthotopic NMIBC models could be used to assess pharmacokinetics, biodistribution, safety, and efficacy following intravesical administration, with the goal of confirming tumor-specific accumulation and localized immune activation while minimizing systemic toxicity. In parallel, synergistic combination strategies will be explored, particularly with immune checkpoint inhibitors (e.g., anti-PD-1/PD-L1), based on the ability of targeted TLR9 activation to convert immunologically “cold” tumors into “hot” ones. Combinations with standard treatments such as mitomycin C or radiation should also be evaluated to determine whether NANO:BICs can amplify the immunogenic cell death elicited by these treatments. Together, these future studies aim to generate the preclinical data required for clinical translation of NANO:BICs as a targeted immunotherapy for NMIBC, addressing limitations of current therapies such as BCG.

### Limitations

While our findings establish a strong proof-of-concept for PSCA-targeted NANO:BICs, it is important to acknowledge the limitations of the present study, which also define the critical next steps for this research. First, our experiments were conducted exclusively using *in vitro* cell line models. While these models are essential for initial validation of the targeting mechanism and cellular uptake in a controlled environment, they are a simplification of the clinical reality. Cancer cell lines lack the complex three-dimensional architecture, cellular heterogeneity, and, most importantly, the rich tumor microenvironment (TME) of an actual tumor. The TME, with its intricate network of stromal cells and infiltrating immune cells, can present physical barriers to nanoparticle penetration and contains immunosuppressive elements that may counteract or *vice versa* improve the intended therapeutic effect. Therefore, the promising results observed here must be validated in more complex models. Second, this study lacks *in vivo* validation. The successful translation of any nanomedicine from the bench to the clinic is contingent upon its performance in a living system. Future animal studies could be the next step to investigate the pharmacokinetics, biodistribution, safety profile, and, ultimately, the therapeutic efficacy of intravesically administered NANO:BICs. Such studies will be crucial to determine if the enhanced *in vitro* uptake translates to preferential tumor accumulation *in vivo* and whether our platform can elicit a robust anti-tumor immune response leading to tumor regression in an orthotopic bladder cancer model. These future investigations, which are currently being planned, will build directly upon the foundational work established in this manuscript.

## Data Availability

The raw data supporting the conclusions of this article will be made available by the authors, without undue reservation.
