# Supplementary material for: PSCA-directed nanosized bio-immune conjugates (NANO:BICs) enable selective uptake of TLR9 agonists in bladder cancer cells
Source: Front Oncol. 2026 Apr 28;16:1739618. doi: 10.3389/fonc.2026.1739618 (PMC13160899; doi:10.3389/fonc.2026.1739618)
Supplement: Supplementary file 1 [file DataSheet1.docx]

**Supplementary material**

**Supplementary methods**

*Physicochemical characterization of NANO:BICs*

*Protein extraction and* *Western Blot (WB)*

To determine the optimal conjugation ratio between NA and the biotinylated scFvs, various molar ratios of scFv (scFv(AM1)-KiBAP and scFv(MR1.1)-KiBAP) and NA were incubated together (Supplementary Figure 1 and Supplementary Figure 2). The resulting conjugates were then analyzed by Western blot to detect conjugate formation via the C-terminal c-Myc epitope of the scFv.

The samples were prepared for electrophoresis by adding 4x LDS sample buffer (Thermo Fisher Scientific) and denaturing at 90°C for 10 minutes. The protein complexes were then separated by molecular weight by SDS-PAGE on a NuPAGE 4-12% Bis-Tris gel (Thermo Fisher Scientific) and subsequently transferred to a nitrocellulose membrane with an iBlot Dry Blotting System (Thermo Fisher Scientific), following the manufacturer's instructions. To detect the scFv, the membrane was probed with a primary antibody targeting the c-myc epitope tag (Invitrogen, R950-25), followed by an HRP-conjugated secondary antibody (Sigma-Aldrich, A0185). Protein bands were visualized using Pierce ECL Western Blotting Substrate (Thermo Fisher Scientific). The resulting chemiluminescent signal was imaged using a MicroChemi 4.2 system (Biostep). Molecular weight estimation was performed using the pre-stained protein ladder Magic Mark XP Western Standard (Thermo Fisher Scientific).

*Multiparameter Nanoparticle Tracking Analysis*

To determine the size and zeta potential of the scFv(AM1)-BAP-loaded NANO:BIC, we used a Zetasizer Ultra Red (Malvern Panalytical) for multiparameter nanoparticle tracking analysis, following the manufacturer’s protocols. NANO:BIC were used in a concentration of 0,5 µM and assembled by two consecutive complexation steps. First, scFv (50 pmol) and NA (50 pmol) were incubated for 30 min at room temperature. ODN (100 pmol) were added to the resulting complex and incubated for another 30 min at room temperature. Data was analyzed using the manufacturer’s software (ZS XPLORER 3.1.0.64).

*Flow cytometry staining*

For the staining of the surface receptor PSCA two samples were analyzed: one sample being stained and one sample serving as control only stained with a fluorochrome-labeled secondary antibody. Controls without primary antibody were used to measure the amount of non-specific binding and background signal of the secondary antibody. First the cells were harvested and 2x10^5^ cells were transferred into FACS-tubes. Then 500 µl PBS-BSA (0.5 %) were added to the cells. The stained sample was then centrifuged at 4 °C for 5 minutes at 300 g and the excess supernatant was discarded. 0.2 µg of the scFv(h-AM1)-KiBAP was added, and the sample was incubated at 4 °C for 1 h. Then 500 µl 0,5 % BSA-PBS were added to the cells. Both the sample and the control were then centrifuged at 4 °C for 5 minutes at 300 g and the excess suspension liquid was discarded. The secondary Fluorescein (FITC)-conjugated AffiniPure Sheep Anti-Mouse IgG (H+L) antibody (Jackson ImmunoResearch) was added to both in a dilution of 1:50 and the cell suspensions were mixed thoroughly. The sample and control were incubated at 4 °C for 30 minutes. In another washing step 500 µl 0.5 % BSA-PBS was added to both the sample and the control, which were then centrifuged at 4 °C for 5 minutes at 300 g and the excess suspension liquid was discarded. Then, 200 µl of 0.5 % BSA-PBS was added and the samples were analyzed using a MACSQuant Analyzer 10 (Miltenyi Biotec). The same procedure was used for the staining of the surface receptor EGFRvIII with the usage of the scFv(MR1.1)-KiBAP.

*Script for the automatic evaluation of fluorescence microscopy*

Script for the automatic evaluation of fluorescence microscopy image files with WGA staining in channel 1, FITC staining in channel 2 and DAPI staining in channel 3. The code was opened and executed as a macro in the ImageJ Macro (IJM) language. Comments are introduced with “//” in the IJM language and serve to explain the subsequent code not altering it.

//Opens a window where the user must choose the input folder

inputDir1= getDirectory("Choose input directory");

//Opens a window where the user must choose the output folder

outputDir = getDirectory("Choose output directory");

//Counts the number of images contained in the input folder

fileList1 = getFileList(inputDir1);

//Opens a loop that repeats itself for every picture in the input folder

for (i = 0; i < fileList1.length; i++) {

//Assigns variable i to the image

file1 = fileList1[i];

filex = inputDir1 + file1;

//Opens image i

run("Bio-Formats", "open=[filex] autoscale color_mode=Default open_files rois_import=[ROI manager] view=Hyperstack stack_order=XYCZT");

//Selects DAPI channel (channel 3)

filea = File.getNameWithoutExtension(file1);

tmp = split(filea, " ");

run("Stack to Images");

selectWindow(tmp[0] + "-0004");

//Prepares DAPI-channel for analysis

run("Gaussian Blur...", "sigma=4 slice");

run("Auto Threshold", "method=Huang2 white");

run("Watershed", "slice");

//Counts cell nuclei in the image

run("Analyze Particles...", "size=50.00-Infinity show=Outlines display exclude summarize slice");

//Saves the resulting image as a tif-file

outName = File.getName(file1) + "_cell count";

saveAs(".tif", outputDir + " " + outName + ".tif");

close(" " + outName + ".tif");

//Saves the cell-nuclei-count as an excel-file

selectWindow("Summary");

saveAs("Results", outputDir + File.getName(file1) + "_cell count.xls");

close(File.getName(file1) + "_cell count.xls");

//Closes the DAPI-channel window

selectWindow(tmp[0] + "-0004");

close();

//Empties the results window

run("Clear Results");

//Closes the ROI manager window

selectWindow("ROI Manager");

run("Close");

//Creates ROI from the WGA-channel and applies it to the FITC-channel

selectWindow(tmp[0] + "-0001");

run("Gaussian Blur...", "sigma=4");

run("Auto Threshold", "method=MinError(I) white");

run("Create Selection");

roiManager("Add");

selectWindow(tmp[0] + "-0003");

//Measures the WGA-channel selection in the FITC-channel

roiManager("Select", 0);

run("Measure");

//Saves the results as an excel-file

selectWindow("Results");

saveAs("Results", outputDir + File.getName(file1) + "_fluorescence result.xls");

//Closes the results and the ROI manager window

close("Results");

close("ROI Manager");

//Saves the selection of the WGA-channel as a tif-file

selectWindow(tmp[0] + "-0001");

saveAs(".tif", outputDir + " " + outName + "_fluorescence treshold.tif");

close();

//Closes all windows

close("*");

//Ends the loop

}

**Supplementary results**

Supplementary Figure 1:


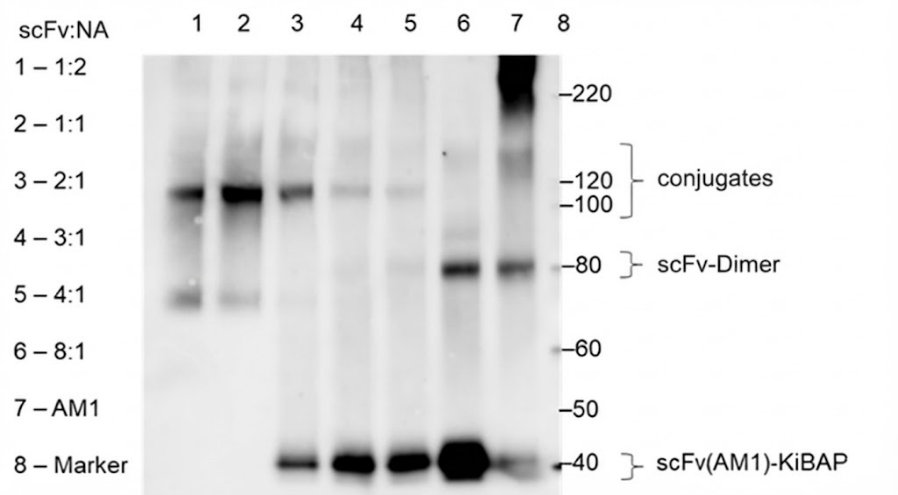


Supplementary Figure 1: Western blot to detect the most **efficient conjugation ratio** of NeutrAvidin and scFv(AM1)-KiBAP. The ratios shown correspond to the ratio of single-chain antibody to NeutrAvidin. The samples were mixed with 4xLDS buffer and incubated at 90°C, applied to a NuPAGE 4-12% Bis-Tris gel, blotted onto a nitrocellulose membrane, and then detected with antibodies against the c-myc epitope. Detection was performed using HRP-conjugated secondary antibodies.

Supplementary Figure 2:


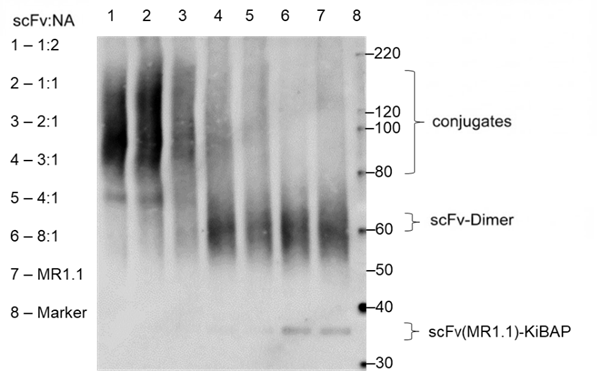


Supplementary Figure 2: Western blot to determine the most **efficient conjugation ratio** of NeutrAvidin and scFv(MR1.1)-KiBAP. The ratios shown correspond to the ratio of single-chain antibody to NeutrAvidin. The samples were mixed with 4xLDS buffer and incubated at 90°C, applied to a NuPAGE 4-12% Bis-Tris gel, blotted onto a nitrocellulose membrane, and then detected with antibodies against the c-myc epitope. Detection was performed using HRP-conjugated secondary antibodies.


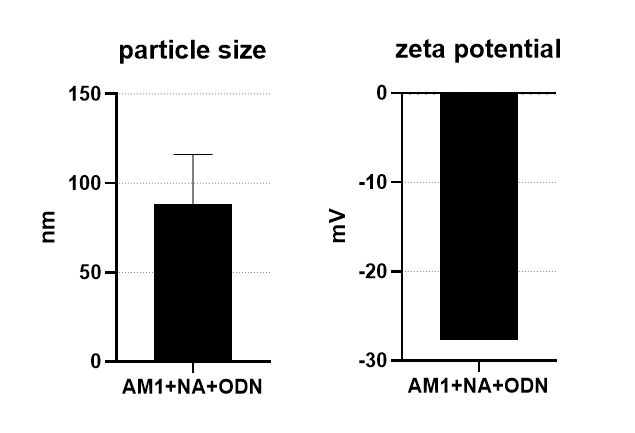


Supplementary Figure 3: **Physicochemical characterization of NANO:BIC complexes**. The formulation, consisting of AM1, NA, and ODN, was characterized by its hydrodynamic diameter (left) and surface charge (right). Mean ± SD

Supplementary Figure 4:

SW780, EGFRvIII stained

SW780, PSCA stained

Supplementary Figure 4: **Quantitation of the surface receptors PSCA (left) and EGFRvIII (right) on SW780 cells using flow cytometry**. The cells were labeled with a specific primary antibody which was detected with a FITC-labeled secondary antibody. Cells stained only with the secondary antibody and their fluorescence in the FITC channel (black curve) served as a control. The differences in the FITC intensity of the two samples were used to determine the percentage of cells expressing the analyzed protein.

Supplementary Figure 5:

HekBlue-hTLR9-PSCA

HekBlue-hTLR9

Supplementary Figure 5: **Staining of the surface receptor PSCA using flow cytometry of the cell lines HEK-Blue^TM^hTLR9 (left) and HEK-Blue^TM^hTLR9-PSCA (right).** The cells were labeled with a specific primary antibody which was detected with a FITC-labeled secondary antibody. Cells stained only with the secondary antibody and their fluorescence in the FITC channel (black curve) served as a control. The differences in the FITC intensity of the two samples were used to determine the percentage of cells expressing the analyzed protein.

Supplementary Results

Western blot analysis was used to assess conjugation efficiency by monitoring molecular weight shifts (Supplementary Figure 1/Supplementary Figure 2). Successful assembly of scFv(AM1)-KiBAP-NA complexes at the expected molecular masses of approximately 120 kDa was indicated by the disappearance of free scFv bands (40 kDa) at molar ratios between 1:1 and 2:1. Based on the maximum signal intensity for conjugates and minimal residual free scFv(AM1)-KiBAP, a 1:1 ratio (as seen in lane 2) was identified as optimal for NANO:BIC assembly. Successful conjugation of scFv(MR1.1)-KiBAP was confirmed by the appearance of high-molecular-weight bands between 80 kDa and 220 kDa, representing various scFv-NA conjugate species. For the scFv(MR1.1)-KiBAP a 4:1 molar ratio was selected for subsequent experiments, as it provided a robust yield of high-molecular-weight conjugates with minimal residual monomeric scFv. To evaluate the physical properties of the AM1+NA+ODN ternary complexes, we performed size and zeta potential measurements (Supplementary Figure 3). The complexes exhibited a mean particle size of 88 ± 27 nm, which is within the optimal range for cellular uptake via endocytosis. Furthermore, the zeta potential was determined to be -27.6 mV, suggesting that the incorporation of ODN contributes a significant negative surface charge, which limits unspecific interactions with sialic acids of cell membranes.
